# Prostate cancer and cardiovascular disease: Racial/ethnic disparities and role of chronic toxic stress/allostatic load

**DOI:** 10.1016/j.ahjo.2024.100397

**Published:** 2024-04-20

**Authors:** Harikrishnan Hyma Kunhiraman, Avirup Guha

**Affiliations:** Cardio-Oncology Program, Medical College of Georgia at Augusta University, Augusta, GA, USA

**Keywords:** Cardio-oncology, Disparities, Prostate cancer

## 1 Introduction

The interconnection between prostate cancer (PC) and cardiovascular disease (CVD), focusing on their shared link of chronic toxic stress was presented at the inaugural UF Health Cardio-Oncology Symposium, “Emerging Topics in Cardio-Oncology.”

## 2 Background

PC is the most diagnosed cancer in men and the second leading cause of cancer death in men, behind only lung cancer [1]. Over the 10-year period after PC diagnosis, CVD accounts for 32 % of deaths in men with PC, whereas PC only accounts for 20 % [2]. PC disproportionately affects African American men when compared to Caucasian men [3]. Specifically, the incidence of PC among African American men is nearly 65 % higher than among Caucasian men, and the mortality of PC is 2.3 times higher [3]. African Americans with PC have a significantly higher risk of mortality due to CVD when compared to Caucasians [3].

Although much research has been performed to identify racial and ethnic differences in PC attributed to ancestry, a significant burden of this difference is related to social determinants of health (SDOH) [4]. The specific SDOHs attributed to PC are financial instability and decreased quality of care [4]. Financial toxicity due to high treatment costs limits treatment adherence as well as the level of treatment offered which negatively affects survival rates [4]. Rural populations with PC have worse outcomes due to further barriers to care including food insecurity, transportation issues, and environmental exposures [4]. The term allostatic load (AL) has been used to characterize this biological burden of stress on the body. In 1993, Bruce McEwen and Eliot Stellar proposed the idea of AL as an extension of the concept of allostasis [5]. Allostasis describes the way in which body systems fluctuate to meet the demands of external stresses – different from the concept of homeostasis, which emphasizes constancy. Chronic stress activates the hypothalamic-pituitary-adrenal axis, sympathetic nervous system and innate immune system, causing the release of corticosteroids and catecholamines, and pro-inflammatory cytokines, respectively. Regarding the innate immune system, chronic stress and associated hormones increase pro-inflammatory cytokines [6]. Studies from Dr. Guha's team suggest that African American men with PC have heightened AL prior to PC diagnosis, and this increased AL increases the risk of CVD by 25 % for every 1-point increase in AL (adjusted hazard ratio [HR] of 1.25 and 95 % confidence interval [CI] of 1.18–1.33) [7].

## 3 Learning points

1. The second highest cause of death in PC is CVD
2. There is a high burden of chronic toxic stress/allostatic load (AL) in men with PC. The burden is higher in Black men.
3. A 1-point increase in AL increases the risk of CVD by 25 %

## Funding acknowledgments

This symposium was funded by the University of Florida Cancer Center, the University of Florida College of Pharmacy Department of Pharmacotherapy and Translational Research, and the University of Florida College of Pharmacy Center for Pharmacogenomics and Precision Medicine.

AG is supported by American Heart Association-Strategically Focused Research Network Grant in Disparities in Cardio-Oncology (#847740, #863620) and the Department of Defense Prostate Cancer Research Program‘s Physician Research Award (#HT94252310158).

## CRediT authorship contribution statement

**Harikrishnan Hyma Kunhiraman:** Writing – review & editing, Writing – original draft. **Avirup Guha:** Writing – review & editing, Writing – original draft.

## Declaration of competing interest

The authors report no conflict of interest relevant to the work presented at this symposium.
